# Water salinity and inundation control soil carbon decomposition during salt marsh restoration: An incubation experiment

**DOI:** 10.1002/ece3.4884

**Published:** 2019-02-10

**Authors:** Faming Wang, Kevin D. Kroeger, Meagan E. Gonneea, John W. Pohlman, Jianwu Tang

**Affiliations:** ^1^ Marine Biological Laboratory The Ecosystems Center Woods Hole Massachusetts; ^2^ USGS Woods Hole Coastal & Marine Science Center Woods Hole Massachusetts; ^3^ Guangdong Provincial Key Laboratory of Applied Botany, South China Botanical Garden Chinese Academy of Sciences Guangzhou China

**Keywords:** carbon dioxide, greenhouse gas, methane, restoration, salt marsh

## Abstract

Coastal wetlands are a significant carbon (C) sink since they store carbon in anoxic soils. This ecosystem service is impacted by hydrologic alteration and management of these coastal habitats. Efforts to restore tidal flow to former salt marshes have increased in recent decades and are generally associated with alteration of water inundation levels and salinity. This study examined the effect of water level and salinity changes on soil organic matter decomposition during a 60‐day incubation period. Intact soil cores from impounded fresh water marsh and salt marsh were incubated after addition of either sea water or fresh water under flooded and drained water levels. Elevating fresh water marsh salinity to 6 to 9 ppt enhanced CO_2_ emission by 50%−80% and most typically decreased CH_4_ emissions, whereas, decreasing the salinity from 26 ppt to 19 ppt in salt marsh soils had no effect on CO_2_ or CH_4_ fluxes. The effect from altering water levels was more pronounced with drained soil cores emitting ~10‐fold more CO_2_ than the flooded treatment in both marsh sediments. Draining soil cores also increased dissolved organic carbon (DOC) concentrations. Stable carbon isotope analysis of CO_2_ generated during the incubations of fresh water marsh cores in drained soils demonstrates that relict peat OC that accumulated when the marsh was saline was preferentially oxidized when sea water was introduced. This study suggests that restoration of tidal flow that raises the water level from drained conditions would decrease aerobic decomposition and enhance C sequestration. It is also possible that the restoration would increase soil C decomposition of deeper deposits by anaerobic oxidation, however this impact would be minimal compared to lower emissions expected due to the return of flooding conditions.

## 1 INTRODUCTION

The carbon (C) burial rate in salt marshes is estimated to be 218 ± 24 g m^−2^ year^−1^, more than 40 times higher than the average soil C burial rate of terrestrial forests (McLeod et al., 2011). One reason for the high C storage rate is that microbial decomposition is relatively slow in marine anaerobic soils, where sulfate reduction is the primary organic matter decomposition pathway (Chambers, Osborne, & Reddy, 2013; Weston, Neubauer, Velinsky, & Vile, 2014). In natural salt marshes, anaerobic conditions are maintained by regular tidal inundation with sea water. This large C storage capacity makes salt marshes important resources for habitat conservation and natural climate solutions (Kroeger, Crooks, Moseman‐Valtierra, & Tang, 2017; Morrissey, Gillespie, Morina, & Franklin, 2014).

A tidal restriction, such as a dike, blocks the flow of sea water to the wetland, resulting in lower salinity, while removal of the restriction can reverse these impacts. How this salinity change affects organic C decomposition is unclear, as previous studies comparing soil decomposition rates along in situ coastal salinity gradients have yielded contrasting results (Chambers et al., 2013; Weston et al., 2014). Craft (2007) observed the highest decomposition rates in the most saline wetlands, and Weston, Vile, Neubauer, and Velinsky (2011) reported accelerated microbial organic matter mineralization following saltwater intrusion into tidal fresh water marsh soils, which was due to increased sulfate reduction (Weston et al., 2014). However, other studies report higher decomposition rates in fresh water tidal wetlands (Quintino et al., 2009; Rejmánková & Houdková, 2006), or no direct relationship between salinity and decomposition rate (Mendelssohn et al., 1999). These inconsistencies highlight the need for a more mechanistic understanding of how salinity affects decomposition. In this study, we use laboratory experiments to isolate the effect of salinity on C decomposition rate and avoid the numerous confounding variables affecting in situ decomposition rates (Chambers, Guevara, Boyer, Troxler, & Davis, 2016; Chambers et al., 2013; Weston, Dixon, & Joye, 2006).

In addition to changing salinity, alteration of marsh hydrology by either building or removing a restriction to tidal exchange has important consequences for C decomposition. For example, in New England, diking reduces or eliminates the 1–2 m semidiurnal tidal range upstream of restrictions (Steever, Warren, & Niering, 1976). As a result, the average water table in the marsh drops from about mean high water to mean sea level (Portnoy, 1999). These changes in water level have a critical influence on coastal wetland biogeochemistry because water table is the primary control on the balance between aerobic and anaerobic respiration (Lewis, Brown, & Jimenez, 2014). The rate of microbial respiration in soil depends primarily on the availability of O_2_ and C in the soil, and on soil temperature and soil moisture, although respiration may also be inhibited when soil water content is either too high or too low (Linn & Doran, 1984). When organic‐rich salt marsh sediments are drained, O_2_ diffuses deeper into the sediment column, stimulating oxic respiration and enhancing decomposition rates and CO_2_ flux out of the soil (Chivers, Turetsky, Waddington, Harden, & McGuire, 2009; Han et al., 2015; Jimenez et al., 2012). Multiple studies have demonstrated that a drop in the water table could accelerate the CO_2_ efflux as much as 50 times faster, possibly due to a combination of increased aerobic oxidation and relief from the ionic stress caused by saltwater inundation (Chambers et al., 2013; Krauss, Whitbeck, & Howard, 2012; Moore & Knowles, 1989; Strakova, Penttila, Laine, & Laiho, 2012; Yang et al., 2014). Therefore, the net effect of either dike emplacement or removal on sediment C decomposition in a restricted marsh is highly dependent on the combined effect of water table, salinity, and flooding duration (Portnoy & Giblin, 1997). However, few studies have investigated the effects of inundation cycles on soil organic carbon (SOC) loss in coastal systems (Bartlett, Bartlett, Harriss, & Sebacher, 1987; Chambers et al., 2016; Neubauer, 2013) and fewer have looked at those differences combined with a salinity shift between fresh and saline.

To better understand how tidal restoration affects organic matter decomposition, we set up a series of sediment core incubations and treated them with either sea water or fresh water to simulate the placement or removal of a dike. The water table was also manipulated to simulate flooding changes after diking. The Herring River impounded salt marsh is an ideal setting to conduct this study. When the salt marsh was diked in 1908, the dominant plant species were *Spartina* spp. and *Distichlis spicata*, all of which are C4 plants with average δ^13^C value ranged from −12‰ to −18‰ (Curtis, Drake, & Whigham, 1989; Redfield, 1972; Redfield & Rubin, 1962). Reduced tidal exchange resulted in freshening of the marsh complex and growth of C3 fresh water plant species with an average δ^13^C value of −27‰. As a result, fresh water marsh peat has accumulated above relict salt marsh deposits. We use these differences in the C stable isotope signature of the deeper C4 deposits and overlying C3 peat to quantify the relative contribution of decomposition of either deep or surface C pools (Cheng, Yang, Li, Dou, & Zhang, 2013; Gunina & Kuzyakov, 2014). We hypothesize that (a) sea water flooding cores collected from the currently impounded fresh water marsh would increase porewater salinity and CO_2_ flux and decrease CH_4_ flux compared to a fresh water flooding treatment; (b) flooding salt marsh cores with fresh water would result in higher CH_4_ flux; (c) lowering the water table would increase total CO_2_ flux, as well as expose carbon from deeper within the soil column to decomposition.

## 2 METHODS

### 2.1 Site descriptions

*Stony Brook* salt marsh (41.754354, −70.115629; Elevation: 1.42 m (NAVD88)): Stony Brook is located in Brewster, Massachusetts (MA), USA. The site is dominated by short form *Spartina alterniflora* (over 90% coverage). The water table relative to the sediment surface ranged from −20 cm to 10 cm inundation in 2016. Stony Brook represents a salt marsh (SM) wetland type.

*Herring River* estuary (41.96058, −70.05587; Elevation: 0.36 m (NAVD88)): The 400‐ha Herring River estuarine complex in Wellfleet (MA) is the largest diked wetland system on Cape Cod, MA, USA. Tidal flow to most of the original *Spartina* marsh transitioned to a fresh water system following inlet closures in the eighteenth and nineteenth centuries, and the construction of a dike across the mouth of the main stream in 1908 (Portnoy & Giblin, 1997). The Herring River site utilized in this study is a former salt marsh, but now a variety of fresh water ecosystems, ranging from forests and shrubs to seasonally flooded fresh water marshes dominated by *Typha angustifolia* (L.), with over 90% coverage. Cores were collected in the fresh water *T. angustifolia* marsh. The water table ranged from 2 cm in the early spring to −50 cm in the later summer in 2016. Herring River estuary represents a diked fresh water marsh (FM) habitat.

### 2.2 Lab experiment

The experimental design consisted of a two by two mixed model treatment. In July 2015, four 0–20 cm soil cores from each of the two sites were collected to determine the general soil and porewater properties (Table 1). Soil cores were sectioned into 0–10 and 10–20 cm layers. Each interval was weighed, and the soil moisture was determined by weight loss after drying a subsample at 105°C for 24 hr. Soil organic matter (SOM) was then measured by the mass loss of ignition (LOI) method (Allen, 1974). The soil properties of the two sites are shown in Table 1.

**Table 1:** General marsh sediments and porewater properties by wetland types

Variables	Layers	fresh water marsh	Salt marsh
Bulk density (g/cm^3^)	0–10 cm	0.15^b^ ± 0.01	0.42^a^ ± 0.01
	10–20 cm	0.21^b^ ± 0.01	0.46^a^ ± 0.01
SOM (%)	0–10 cm	97.4^a^ ± 0.94	39.8^b^ ± 1.18
	10–20 cm	72.2^a^ ± 4.05	37.9^b^ ± 4.47
Salinity (ppt)		0.1^b^ ± 0.1	26.8^a^ ± 0.9
pH		5.58^b^ ± 0.18	7.17^a^±0.06
Redox		34.2^a^ ± 15.3	−271^b^ ± 26.6
DOC (mg/L)		67.2 ± 18.7	48.6 ± 14.0

Note

Fresh water marsh sediments were from the Herring River Basin, Wellfleet, MA; Salt marsh cores were from Stony Brook, Brewster, MA. The different superscript letters in each soil layer indicate that there are significant difference among treatments (One‐way ANOVA: *p* < 0.05), while shared same letters indicate no significant difference.

In November 2015, eight intact soil cores were collected from each of the two wetland types (diked fresh water marsh (FM) and salt marsh (SM)) in 60 cm long 10 cm outer diameter (o.d.) clear polycarbonate tubes. The length of the soil cores in each tube was about 40 cm. Cores were collected in winter to reduce the influence of live plant root respiration on the soil CO_2_ flux. All eight soil cores from each of the two sites were collected within a 2‐m × 2‐m plot to minimize heterogeneity between cores. Aboveground dead vegetation was removed by clipping, the cores were capped on top and bottom, and then transported back to the laboratory.

In the laboratory, the bottom of each core was sealed with a plastic cap, and a drain hole was added with a stopcock. The 16 cores, eight from salt marsh and eight from fresh water marsh, were treated with two types of water (fresh deionized (DI) water and sea water, hereafter FW and SW, respectively). Therefore, there were four replicates for each treatment (SM + SW, SM + FW, FM + SW, and FM + FW). The SM + FW treatment simulated the impact of restricting tidal exchange and freshening salt marsh sediments, while the FM + SW treatment simulated the restoration of tidal exchange if the restriction was removed. The remaining two treatments (FM + FW and SM + SW) act as control treatments and represent no change in salinity from existing conditions. The cores were left open to the atmosphere at the top and were stored in a controlled environment with an ambient temperature of approximately 21°C. The soil cores were acclimated for one week before the incubation began to minimize the impact of field sampling disturbance on the gas flux.

At the beginning of the incubation, sea water or fresh water was added to a level 2 cm above the soil surface of each core. The 2‐cm deepwater column mimics tidal inundation in the salt marsh and seasonal flooding in the fresh marsh. The water was refilled as needed during the incubation period to maintain the water level in the core. The sea water was collected from the Marine Biological Laboratory's docks in Woods Hole, MA and then filtered it using 25 mm GF/F Swinex filters. Since both fresh water marsh and salt marsh sediments are more than 50% organic matter and the porewater nutrient concentrations are high, we assumed that DI water addition would not lead to nutrient limitations. We measured gas flux before water was added to the cores to simulate inundation at timepoint 0. Water level was then adjusted to flooded conditions and gas flux was measured at 0.5,1, 3, 5, 7, 9, 11, 14, 19, 23, 30 days. After 30 days of inundation, we drained the cores until the water table was 20 cm below the core surface, representing the general water table of fresh water marshes in the late summer. All soil cores were incubated for 30 more days after draining, and the gas flux was measured at day 2, 6, 9, 12, 15, 17, 24, 26, and 30 after draining (i.e., 32, 36, 39, 42, 45, 47, 54, 56, 60 days from the start of the experiment).

To measure gas flux, a plastic chamber was placed on top of the soil core tube and sealed with a rubber ring. CO_2_ and CH_4_ concentrations in the headspace were recorded at 1 Hz over a five‐minute period where the CO_2_ concentration in the chamber was rising steadily. During the first and the last three weeks incubation, the gas flux from the cores was measured using a G‐2301*f* Picarro CO_2_, CH_4_, and H_2_O gas analyzer (Picarro Inc. Santa Clara, CA, USA). Gas flux was calculated from the linear slope of CO_2_ and CH_4 _concentrations over time point according to:

(1) F=(dc/dt)×(1/V0)×(P/P0)×(T0/T)×(V/S)

where *F *is the flux rate, d*c*/d*t *is the slope of the CO_2_ or CH_4_ concentration versus time, *V*0 is the CO_2_ or CH_4_ molar volume under standard conditions (i.e., 22.4 L/mol), *P *is the air pressure in the laboratory, *P*0 is the standard air pressure, *T *is the air temperature during each measurement, *T*0 is the standard temperature, *V *is the head space volume, including the tubing volume, and *S* is the soil surface area of the soil core. The calculation was conducted in Matlab 2016a (The MathWorks, Natick, MA, USA). The gas concentrations and δ^13^C values of CO_2_ and CH_4_ flux were measured using a Picarro G‐2201*i* gas analyzer (Picarro Inc. Santa Clara, CA) for both the flooded and drained treatments in the fourth and fifth week, respectively. The CH_4_ and CO_2 _stable carbon isotope values (expressed as *i*CH_4_ and *i*CO_2_, respectively) measured with the G‐2201i gas analyzer were corrected using a slope and offset correction based on a linear best‐fit regression between the measured values and standards of known isotopic content (Pohlman et al., 2017) :

(2) $${\text{Data}}_{\text{corrected}}=\text{Slope}\times {\text{Data}}_{\text{measured}}+\text{Offset}$$

The slopes and offsets for the calibration were determined from Isometric gas standards with δ^13^C values of −66.5‰, −38.3‰, and −23.9‰ for CH_4_, and secondary CO_2_ standards with δ^13^C values of −42.9‰, −26‰, and −1.6‰ (±0.5‰). The isotope standards were analyzed once a week. The δ^13^C of the CH_4_ and CO_2 _flux was determined from measurements taken 5–10 min after placing the chamber on the soil core to accumulate sufficient gas for δ^13^C analysis and calculated according to:

(3) $$\delta {\text{C}}^{13}{\;\text{of CO}}_{2}\;\text{flux}=\left({\text{C}}_{\text{end}}\times \delta {\text{C}}_{\text{end}}^{13}-{\text{C}}_{\text{initial}}\times \delta {\text{C}}_{\text{initial}}^{13}\right)/\left({\text{C}}_{\text{end}}-{\text{C}}_{\text{initial}}\right)$$

where C_end _is the mean CO_2 _concentration of in the last 60 s of measurement, C_initial_ is the mean CO_2_ concentration of in the first 60 s, δC^13^_end_ is the mean δC^13^ of CO_2_ in the last 60 s, and δC^13^_initial _is the mean δC^13^ of CO_2_ in the first 60 s. A similar calculation was conducted for the δC^13 ^of the CH_4 _flux.

Methane flux data are only available from the flooded incubation experiment due to interference of the CH_4_ signal by an unidentified compound (perhaps NH_3 _due to the high soil total N concentration (Table 2) or hydrogen sulfide). Therefore, CH_4_ data are only reported for the first 30 days of the flooded experiment.

**Table 2:** Sediment carbon and nitrogen concentration and isotope signature after incubation

Soil layers	Treatments	Soil C (%)	δ^13^C (‰)	Soil N (%)	C/N
Top	FM + FW	45.40^a^ ± 0.46	−26.45^a^ ± 0.03	3.33^a^ ± 0.08	13.66 ± 0.31
Top	FM + SW	38.88^ab^ ± 1.13	−26.98^a^ ± 0.21	2.69^b^ ± 0.12	14.52 ± 0.35
Top	SM + FW	20.55^c^ ± 1.54	−16.63^b^ ± 0.14	1.48^c^ ± 0.09	13.91 ± 0.45
Top	SM + SW	18.45^c^ ± 0.83	−16.18^b^ ± 0.17	1.30^c^ ± 0.07	14.18 ± 0.31
Bottom	FM + FW	30.08^a^ ± 4.18	−15.15 ± 0.36	1.99^a^ ± 0.23	14.93^a^ ± 0.69
Bottom	FM + SW	30.08^a^ ± 1.56	−16.03 ± 0.68	2.00^a^ ± 0.13	15.10^a^ ± 0.51
Bottom	SM + FW	18.60^b^ ± 2.48	−18.10 ± 1.04	1.38^b^ ± 0.16	13.38^b^ ± 0.35
Bottom	SM + SW	17.40^b^ ± 1.84	−17.10 ± 0.67	1.36^b^ ± 0.10	12.71^b^ ± 0.65

Note

The different superscript letters in each soil layer indicate that there are significant differences among treatments (Fisher's LSD, *p* < 0.05), while shared same letters indicate no significant difference. Top is 0–5 cm soils and Bottom is 35–40 cm soils. FM: fresh water marsh; FW: fresh water; SM: salt marsh; SW: sea water.

After the incubation, soil samples from the surface (0–5 cm) and bottom of each core (35–40 cm) were collected. The total soil C and N concentrations, as well as the δ^13^C were measured on an Isoprime 100 IRMS (Isoprime Ltd., Cheadle Hulme, UK). The rate of SOC loss was estimated by measuring the major pathways of organic C loss, including CO_2_ production (aerobic and anaerobic decomposition) and CH_4_ production (methanogenesis).

In the fresh water marsh soil cores, OC from deeper profile had a C4 plant origin, while surface organic matter was derived from C3 plants, each having a unique stable carbon isotope signature. Thus, the δ^13^C values of the CO_2_ flux and soil C allowed us to calculate the proportions of surface C (*f*_C3_, C derived from recent C3 plants) and deep C (*f*_C4_, the organic C from C4 plants) that contributed to CO_2_ produced during the incubations using the following mass balance equation (Cheng et al., 2013; Del Galdo, Six, Peressotti, & Cotrufo, 2003):

(4) $$f_{\text{C}3}=\frac{\delta \text{C}4-\delta {\text{CO}}_{2}}{\delta \text{C}4-\delta \text{C}3}$$

where δC4 is the δ^13^C value of core bottom soil C, δC3 is the core surface δ^13^C value, δCO_2_ is the δ^13^C values in respired CO_2_ flux, and *f*_C3_ is the fraction of CO_2_ from surface sediments. *f*_C4_, the fraction of CO_2_ sourced from deeper sediments, then equals 1 − *f*_C3_.

Porewater samples were collected at the bottom of each soil core using a 50 ml syringe at the beginning of the experiment prior to water addition treatment, and on day 14, 30, 44, and 60. In two cores (one SM + FW and one SM + SW replicate), the drain became blocked during the drained incubation, so water samples were not collected. Porewater pH (using a Spectrum FieldScout SoilStik pH meter, Spectrum Inc. Aurora, IL), redox (using a Spectrum FieldScout SoilStik electrode meter, Spectrum Inc. Aurora, IL), and salinity (using a refractometer) were measured. The porewater was then filtered through a 47 mm GF/F filter. Once filtered, dissolved organic carbon (DOC) samples were acidified with 10 μl of HCl for storage. DOC samples were run on a total organic carbon analyzer (OI Analytical, Aurora 1030c).

### 2.3 Statistical analysis

As our experiment used a two by two random design, with each treatment replicated four times, we used ANOVA (Analysis of Variance) to assess differences in the initial soil and porewater properties between the two marshes (Table 1). After incubation, the soil C and N parameters were again analyzed with ANOVA (Table 2), followed by a least significant difference (LSD) multi‐comparison. Before ANOVA analysis, the homogeneity of variances was checked with Levene's test, due to the inhomogeneity of variation in soil Redox, non‐parametric analysis was conducted. Linear mixed effects models (LMMs, nmle package in R 3.2.5, Pinheiro, Bates, DebRoy, Sarkar, and R‐Core‐Team, (2016)) were used to examine the effects of treatments on CO_2_ and CH_4_ flux, with source of marsh soil (i.e., from the salt marsh or fresh water marsh), water category (i.e., fresh water or sea water), source marsh*water category interaction, and inundation treatment (i.e., flooded or drained) the fixed effects, and replicates and sampling time grouped within replicates the random effects. In the CH_4_ flux analysis, marsh sediment provenance, water treatment, and their interaction were fixed effects, while replicates and sampling time within replicates were random effects. For soil water DOC, pH, redox, and salinity, the marsh sediments provenance, water treatment, sediments*water interaction, and inundation treatment were regarded as fixed effects, and replicates and sampling time grouped in replicates were random effects. Finally, in the analysis of the δC^13 ^of CO_2_ and CH_4_ flux, and the fraction of C sourced from surface or deeper sediments, marsh sediment provenance, water treatment, sediments*water interaction, and inundation treatment were fixed effects, and replicates were the random effect. Results are reported as significant at *p* < 0.05. All data analyses were performed using R language 3.2.5.

## 3 RESULTS

### 3.1 Soil and water properties

**Figure 1:**
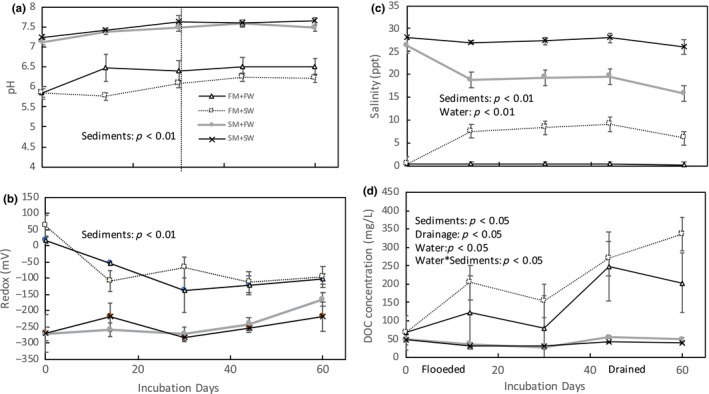
Sediment porewater pH, oxidation and reduction potential (redox), salinity and dissolved organic carbon (DOC) during the two‐month incubation (Error bars indicate standard error for each treatment, *n* = 4). FM: fresh water marsh; FW: fresh water; SM: salt marsh; SW: sea water. Dotted lines separate the flooded condition and drained condition

Based on additional cores collected at each site, salt marsh soil had 2–3 times higher soil bulk density (BD) than the fresh water marsh (Table 1, One‐way ANOVA: *p* < 0.05). As observed in other wetlands, there was an inverse relationship between soil organic matter content and bulk density. Fresh water marshes had higher soil C (30%–45%) compared to salt marsh soils (18%–21%) (Tables 1 and 2). Similar to soil C, porewater DOC was also higher in the fresh water marsh than the salt marsh (Table 1). The in situ porewater salinities were 0.1 and 26.8 ppt for the fresh water marsh and salt marsh, respectively. Porewater pH was higher in the salt marsh than fresh water marsh, while the redox potential was reversed, with lower redox in the salt marsh than the fresh water marsh. The addition of sea water increased fresh water marsh salinity from 0.1 ppt to 8.12 ppt, while the addition of fresh water decreased the salt marsh salinity from 26.6 ppt to 18.6 ppt (Figure 1c).

After the incubation experiment, the sediment cores were subsampled at 0–5 and 35–40 cm and analyzed. The top soil (0–5 cm) of the fresh water marsh sediments (FM) had twice the C concentration (38.9%–45.4%, Table 2) compared to the salt marsh sediments (SM, Fisher's LSD: *p* < 0.05). The high FM %C results from plant organic matter dominating surface soils. Soil %C decreased with soil depth in FM (Table 2), reaching 30% in the bottom section (35–40 cm). However, the SM soil C% did not decrease with depth. Total N concentration followed a similar pattern as soil C between treatments and soil depth, so, as a result, there was no difference in soil C/N ratio (13.7–14.5) among marshes in surface soils (Table 2). In the bottom soil section, the FM (14.9–15.1) had significantly higher C/N ratio than SM (12.7–13.4). The δ^13^C ranged from −27.0‰ to −26.5‰ in the surface soil of FM, but was much higher in SM soils and FM bottom soils (ranging from −15.5‰ to −18.1 ‰, Table 2, Fisher's LSD: *p* < 0.05).

### 3.2 Soil porewater properties and gas emissions

Porewater collected from salt marsh sediments had a significantly higher pH (Sediments effect: *p* < 0.05) than the fresh water marsh cores, and the addition of either fresh water or sea water did not alter this pattern. Sea water addition decreased the pH in the FM+SW treatment compared to the FM + FW reference (Figure 1a). Porewater redox varied significantly between the two marsh sediments, with no effect from either water salinity or inundation level, with salt marsh porewater Rh lower than in the fresh water marsh (*p* < 0.05, Figure 1b). Both marsh sediment source and the salinity of the water added to the incubation cores greatly affect the porewater salinity (*p* < 0.05 and *p* < 0.01, respectively). Soil porewater DOC varied between marsh soil source and inundation level, but not among water types (Figure 1d). The fresh water marsh usually had higher DOC than the salt marsh, consistent with the redox potential. The significant effect of marsh × water interaction indicated that exposing sediments fresh salt or fresh water marshes to porewater with a different salinity than they experienced in situ increases DOC concentrations. This effect was more pronounced in the fresh water marsh (Figure 1d). Porewater DOC increased during the drained incubation period compared to the earlier flooded treatment (*p* < 0.05, Figure 1d).

**Figure 2:**
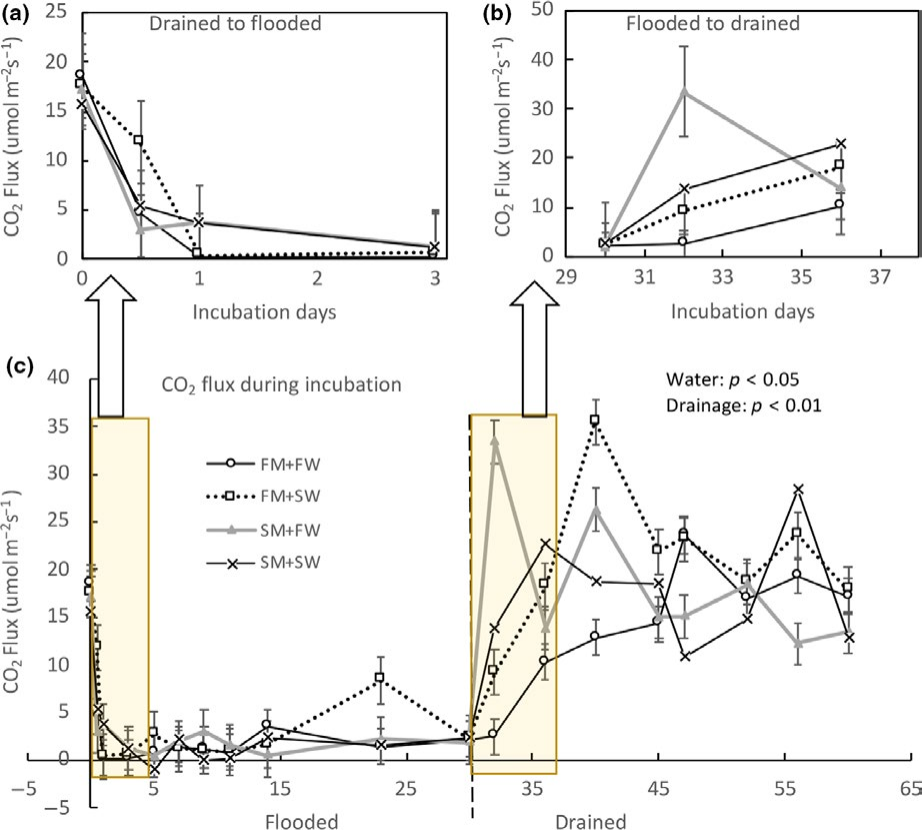
CO_2_ flux in the incubation experiment with different marsh sediments and water treatments. At day 60 the cores shifted from flooded to drained conditions. (Error bars indicate one standard error, *n* = 4). FM: fresh water marsh; FW: fresh water; SM: salt marsh; SW: sea water

**Figure 3:**
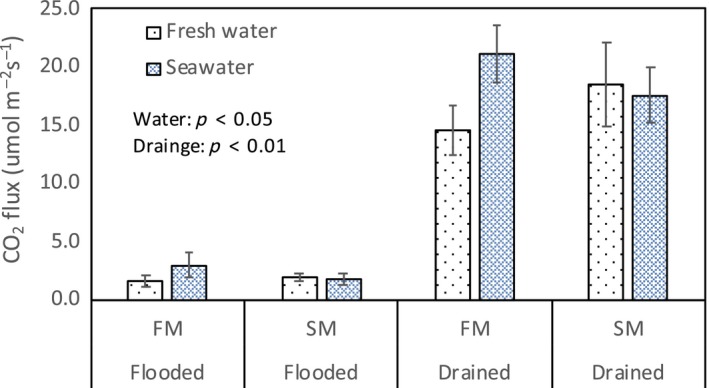
The mean CO_2_ flux was calculated from all flux data for each treatment (core# *n* = 4, measurement *n* = 9 during all sampling events) during either flooded or drained conditions, for fresh water or salt marsh sediments with different water treatments and inundation levels (error bar indicates 1 standard error). FM: fresh water marsh; SM: salt marsh

The inundation treatment was an important control on soil CO_2_ flux (Figures 2 and 3). In both sediment treatments, CO_2_ flux was significantly lower during the flooded treatment (water table: 2 cm) than the drained treatment (water table: −20 cm) (Drainage effect: *p* < 0.01, Figures 2 and 3). After the first day of flooded conditions, CO_2_ flux decreased rapidly in both fresh and sea water (Figure 2a). Initially, CO_2_ flux was over 15 µmol CO_2_ m^−2^ s^−1^ before any water addition, potentially due to continuing evasion of CO_2_ from the initial equilibration period before the water level was adjusted to the treatment level. After this period, fluxes were less than 5 µmol CO_2_ m^−2^ s^−1^ in all treatments. During the one‐month flooding incubation, the soil CO_2_ flux was much lower than the following one‐month drained incubation (Figures 2c and 3). There was a large initial increase in soil CO_2_ flux after the cores were drained (Figure 2b). However, after one week, the rate of soil CO_2_ flux largely stabilized (Figure 2b), although a small decrease continued after this time. Overall, the sediment treatment, that is, fresh water marsh or salt marsh, did not effect the CO_2_ flux, while water types and inundation condition had significant effects (*p* < 0.05 and *p* < 0.01, respectively). The interaction of source marsh and water type also significantly affected the soil CO_2_ flux (*p* < 0.05), with higher soil CO_2_ flux measured in the saltwater addition to the fresh water marsh (FM + SW) than the values in the reference FM + FW under both inundation levels (Figure 3), indicating higher microbial anaerobic respiration after saltwater addition.

**Figure 4:**
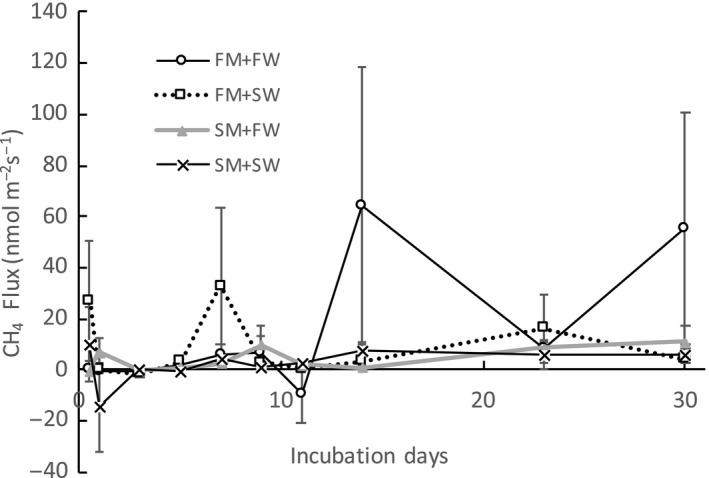
CH_4_ flux in the flooded incubation experiment with different marsh sediments and water treatments (error bar indicates 1 standard error, *n* = 4). FM: fresh water marsh; FW: fresh water; SM: salt marsh; SW: sea water

Due to the high variability of methane flux across replicate soil cores, neither sediment source or water type treatment significantly affected CH_4_ fluxes during flooded conditions. In one of FM + FW core, we recorded over 100 times higher methane emission than the other three cores during some sampling events, which lead to very high error in the FM + FW CH_4_ flux. Overall, there was no consistent methane flux pattern during the 30‐day incubation (Figure 4).

### 3.3 Stable carbon isotopes and the source of soil respiration

**Figure 5:**
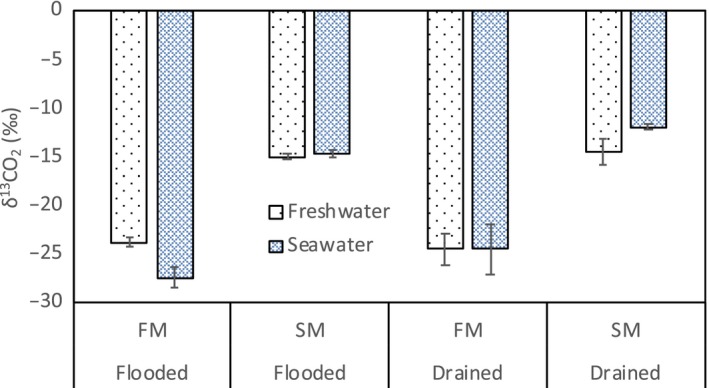
The δ^13^C of the CO_2_ flux under different marsh sediment source, water type, and inundation treatments (error bars indicate standard error, *n* = 4). FM: fresh water marsh, SM: salt marsh

**Figure 6:**
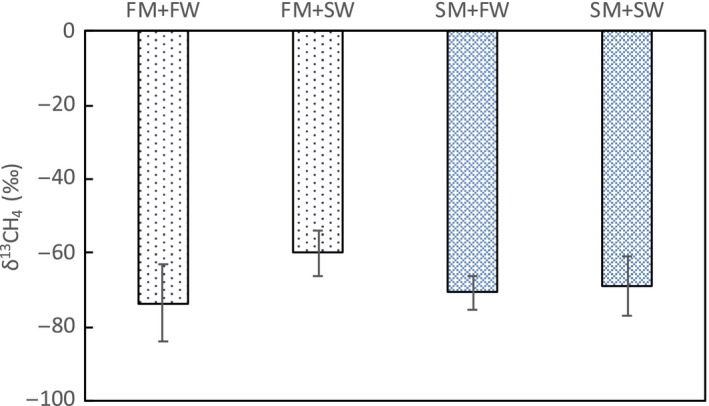
The δ^13^C of the CH_4 _flux for different marsh sediment sources and water salinities during flooded conditions. (CH_4_ isotope measurements from the drained incubations are excluded, see methods). Error bar indicates standard error, *n* = 4). FM: fresh water marsh; FW: fresh water; SM: salt marsh; SW: sea water

The δ^13^C of the CO_2_ and CH_4_ flux was measured twice during the course of the experiment. In the flooded treatment, the mean δ^13^C of the CO_2 _flux in FM + FW was −23.8 ± 0.32‰, which was more ^13^C enriched than the mean value in FM + SW, though the difference was not significant (−27.5 ± 1.97‰, *p* = 0.08). There was no difference between these treatments when drained (Figure 5). The δ^13^C of the CH_4_ flux ranged from −60.0‰ to −73.5‰, with no difference among treatments (Figure 6).

The δ^13^C of the CO_2 _flux was used to find the proportion of CO_2_ flux derived from respiration of either C3 or C4 plant material in the fresh water marsh cores (Table 2). In these cores, the surface sediments have the signature of fresh water marsh vegetation (C3 plants), while organic matter deeper in the soil column is derived from salt marsh vegetation (C4 plants). During flooded and drained fresh water conditions, 23.6%–28.4% of the CO_2_ flux was derived from organic matter deeper in the soil column. In the flooded sea water treatment, most of the respiration (107.0 ± 18.3%, Table 3) was derived from surface organic matter; however, this was not statistically significant due to large variation among soil cores in this treatment. The drained sea water treatment had the greatest amount of CO_2_ flux from the deeper soil organic matter (41.3 ± 8.6%, Table 3). A similar analysis was not possible in the salt marsh cores since the entire soil column was sourced from salt marsh C4 plant material.

**Table 3:** Proportion of CO_2_ flux derived from surface and deep organic matter during flooded and drained conditions in the fresh water marsh sediments

Inundation conditions	Treatments	CO_2_ from deeper C	CO_2_ from surface C
Flooded	FM + FW	23.6 ± 3.0%	76.4 ± 3.0%
Flooded	FM + SW	−7.0 ± 18.3%	107.0 ± 18.3%
Drained	FM + FW	28.4 ± 8.2%	71.6 ± 8.2%
Drained	FM + SW	41.3 ± 8.6%	58.7 ± 23.3%

Note

FM: fresh water marsh; FW: fresh water; SM: salt marsh; SW: sea water.

## 4 DISCUSSION

### 4.1 Soil properties

Salt marsh sediments had higher soil bulk density and lower percent soil organic matter, total C, and total N content than fresh water marsh sediments, likely due to greater inorganic sediment deposition due to periodic high tide flooding, as observed in other salt marshes (Chambers et al., 2013; Craft, 2007; Drake, Halifax, Adamowicz, & Craft, 2015; Morris et al., 2016). In contrast, the fresh water marsh soil consisted of highly decomposed plant material, and usually had lower bulk density and higher soil C and N (Table 2). Furthermore, the surface soil δ^13^C in the fresh water marsh ranged from −27.0‰ to −26.5‰, indicating that soil C derived from the dominant C3 plant *Typha angustifolia*. However, deeper in the soil column (35–40 cm)), the δ^13^C ranged from −15.5‰ to −18.1 ‰, which indicated a C4 plant source. This shift reflects the stratigraphic boundary when tidal flow to the former salt marsh was restricted and the area converted to a fresh water wetland (Portnoy & Giblin, 1997). The variation in δ^13^C signatures of different soil layers provides an opportunity to determine the relative contribution of deep or surface organic matter to respired CO_2_ flux.

### 4.2 CO_2_ and CH_4_ gas flux

The addition of sea water to fresh water marsh sediments (FM + SW) increased organic matter respiration and CO_2_ flux, confirming our first hypothesis. This result is consistent with other studies that found increased microbial decomposition rates after salinity intrusion in fresh water wetlands (Craft, 2007; Weston et al., 2006, 2011). In an incubation experiment, Weston et al. (2006) observed that sulfate reduction became the dominant pathway of organic matter oxidation within two weeks of salinity intrusion, and accounted for >95% of total organic matter mineralization after four weeks. Sulfate reduction also blocked methanogenesis. In this experiment, some measurements resulted in lower methane emission in the FM + SW treatment compared to the FM + FW treatment. These results generally support those of Poffenbarger, Needelman, and Megonigal (2011), who found decreasing CH_4_ with increasing salinity and sulfate concentrations. In the present study, although sulfate reduction was not directly measured, there was an over 80% increase in CO_2_ flux in FM + SW cores compared to FM + FW cores in the flooded treatment, and a 50% increase in the drained treatment. There was also a pronounced increase in porewater DOC concentration after sea water addition to the fresh water marsh cores (Figure 4d). The increased soil decomposition and DOC concentration are likely due to increasing rates of sulfate reduction after sea water was added to the cores with a source of new sulfate.

In contrast, the addition of fresh water to salt marsh sediments had no effect on CO_2_ flux, CH_4_ flux, or porewater DOC concentrations over the course of the experiment. This result is inconsistent with our hypothesis and with the results from a similar laboratory experiment by Chambers et al. (2013), who observed SOC loss increased in salt marsh sediments after pulsed fresh water additions. However, this is likely an artifact of our experimental design and the geochemistry of the fresh water marsh sediments. In Chambers et al. (2013)’s experiment, they added several fresh water pulses to the salt marsh sediment, draining the water several times, ultimately resulting with very low porewater salinity. In the current experiment, the initial porewater was not drained, leaving salinity up to 18 ppt in the SM + FW cores, much higher than that in FM + SW cores even after addition of sea water. Since the salinity remained elevated, it is possible that with the presence of high porewater sulfate concentrations, sulfate reduction remains the dominant pathway of organic matter oxidation in the salt marsh sediment cores, and furthermore inhibits CH_4_ flux. The results are in general agreement with Poffenbarger et al. (2011), since partial reduction in salinity to ~18 ppt did not result in increased CH_4_ flux from salt marsh sediments. This result is relevant to real‐world conditions, since in many cases some portions of restricted or restored marshes may experience only moderate changes in salinity.

The control of water level on soil respiration rates is well documented in fresh water wetlands (Dehedin, Maazouzi, Puijalon, Marmonier, & Piscart, 2013; Laiho, 2006). However, few studies have investigated how changes in salinity and inundation levels impact coastal wetland CO_2_ flux (Chambers et al., 2013). We found that inundation treatment, that is, flooded or drained, had a more pronounced effect on the CO_2_ flux than changes in salinity. As stated above, adding sea water to fresh water cores increased CO_2_ flux by 50%–80% in either flooded or drained treatments, while draining the soil cores emitted nearly 10‐fold higher CO_2_ than the flooded reference cores for both marsh sediments, confirming our third hypothesis. Under drained conditions, O_2_ diffuses deeper within the soil column, enhancing the metabolic activity of soil microorganisms, and promoting CO_2_ flux out of the soil. Moore and Knowles (1989) also found that CO_2_ emissions from completely flooded cores (similar to our flooded treatments in this study) were much lower than emissions from partially flooded cores. Therefore, our results indicate that permanent alteration of marsh hydrology by building and/or remove tidal restrictions has a much more pronounced impact on the rate of C decomposition than does the change of water salinity.

Using natural δ^13^C variability between C3 and C4 plants to investigate the source of respired soil C and CO_2_ flux has been well documented in agriculture and grassland ecosystems (Paterson, Midwood, & Millard, 2009). In the fresh water marsh sediment, the upper layer C3 and deeper layer C4 plant organic matter distribution allowed us to detect the sources of respired CO_2_. In the FM + FW reference cores, over 70% of CO_2_ was derived from C3 plant material in both flooded and drained conditions, indicating that most of the respired C came from surface organic matter. However, the remaining CO_2_ flux came from deeper organic matter, indicating that these soils are potentially still losing relict buried carbon even under completely flooded conditions. However, there are some methodological issues in using this approach to investigate the CO_2_ sources in wetland soils under flooded conditions. If CH_4_ production and associated CO_2_ production from the anaerobic oxidation of methane increased, isotopic fractionation associated with the low δ^13^C signature of biologically generated CH_4_ would lower the δ^13^C of the CO_2_ flux due to remineralization pathway, not organic matter source (Templeton, Chu, Alvarez‐Cohen, & Conrad, 2006). Moreover, sulfate reduction also yields ^13^C‐depleted CO_2_ (fractionation factor of 1.031, Londry and Des Marais (2003)). Both of above processes would result in an overestimation of CO_2_ source from C3 plant material. This could partially explain the very depleted δ^13^C CO_2 _values observed in FM + SW treatments. However, in the drained treatment in this study, the CO_2_ flux was much greater than the flooded condition, so we predict that most of the increased CO_2_ flux was from increased aerobic oxidation processes, with different enzyme processes having similar isotope fractionation factors (Fernandez, Mahieu, & Cadisch, 2003; Paterson et al., 2009). Therefore, the difference in ^13^C abundance in the CO_2_ flux between FM + FW and FM + SW treatments should reflect changes in respired C source in each treatment.

Intact soil cores were used to simulate tidal restoration of a restricted marsh and evaluate the impact of inundation level and soil salinity on C decomposition rates. However, there remain some uncertainties and caveats to consider when applying these laboratory results to field sites. For example, the one‐month incubation period for the flooded and drained treatments may not reflect the in situ long‐term porewater salinity changes, flooding regimes, or microbial community shifts. This is relevant in particular to methane emissions, since the 30‐day incubation may have been too short to allow for shifts in microbial populations, although Edmonds, Weston, Joye, Mou, and Moran (2009) found no changes in microbial community composition of bacteria or archaea after sediment cores had been exposed to sea water for 35 days. In addition, short‐term sea water addition experiments have also resulted in similar flux patterns in sediment incubations (Vizza, West, Jones, Hart, & Lamberti, 2017). Although further observations and modeling are necessary to determine if all of our findings can be replicated under in situ conditions, a mesocosm study in a brackish mangrove documented that increased inundation had a greater impact on the soil microbial community than increased salinity (Chambers et al., 2016). Moreover, a field study in salt marshes has reported decrease in CO_2_ flux following increased inundation (Neubauer, 2013). The findings in this study thus are consistent with these field observations and have some useful information for salt marsh restorations.

### 4.3 Implications for salt marsh restoration

This study suggests that restoration of tidal flow that raises the water level from drained conditions would greatly decrease aerobic decomposition and enhance C sequestration. It is also possible that the restoration of tidal flow increase soil C decomposition of deeper deposits by anaerobic oxidation; however, this impact would be minimal compared to lower emissions expected due to the return of flooding conditions. Specifically, in the case of the Herring River, where these fresh water marsh sediments were collected, we predict restoration of tidal flow would (a) greatly inhibit the aerobic decomposition as water level increased, (b) increase anaerobic oxidation via sulfate reduction as sea water flooding increased, and (c) increase C storage rates as greater inundation reduces soil organic matter remineralization rates. Therefore, salt marsh restoration at this site would yield greater soil C storage.

## CONFLICT OF INTEREST

None declared.

## AUTHORS CONTRIBUTION

FW designed the experiment and conducted the data collection, and wrote the manuscript. JT and KK involved in experiment design, data collection, and manuscript preparation. MG involved in data collection and manuscript preparation. JP involved in isotope data collection and manuscript preparation.

## Data Availability

All data are provided in full in the results section of this paper and will be available at https://datadryad.org (https://doi.org/10.5061/dryad.g333f25) and https://github.com/famingw/marshincubation after the paper is published online.
